# Topical Beta-Blockers and Cardiovascular Mortality: Systematic Review and Meta-Analysis with Data from the EPIC-Norfolk Cohort Study

**DOI:** 10.1080/09286586.2016.1213301

**Published:** 2016-08-23

**Authors:** Claude Pinnock, Jennifer L. Y. Yip, Anthony P. Khawaja, Robert Luben, Shabina Hayat, David C. Broadway, Paul J. Foster, Kay-Tee Khaw, Nick Wareham

**Affiliations:** ^a^ Department of Public Health and Primary Care, University of Cambridge, Cambridge, UK; ^b^ NIHR Biomedical Research Centre for Ophthalmology, Moorfields Eye Hospital and UCL Institute of Ophthalmology, London, UK; ^c^ Department of Ophthalmology, Norfolk and Norwich University Hospital, Norwich, UK; ^d^ MRC Epidemiology Unit, University of Cambridge School of Clinical Medicine, Cambridge, UK

**Keywords:** Beta-blockers, cardiovascular, epidemiology, mortality, topical

## Abstract

***Purpose***: To determine if topical beta-blocker use is associated with increased cardiovascular mortality, particularly among people with self-reported glaucoma.

***Methods***: All participants who participated in the first health check (*N* = 25,639) of the European Prospective Investigation into Cancer (EPIC) Norfolk cohort (1993–2013) were included in this prospective cohort study, with a median follow-up of 17.0 years. We determined use of topical beta-blockers at baseline through a self-reported questionnaire and prescription check at the first clinical visit. Cardiovascular mortality was ascertained through data linkage with the Office for National Statistics mortality database. Hazard ratios (HRs) were estimated using multivariable Cox regression models. Meta-analysis of the present study’s results together with other identified literature was performed using a random effects model.

***Results***: We did not find an association between the use of topical beta-blockers and cardiovascular mortality (HR 0.93, 95% confidence interval, CI, 0.67–1.30). In the 514 participants with self-reported glaucoma, no association was found between the use of topical beta-blockers and cardiovascular mortality (HR 0.89, 95% CI 0.56–1.40). In the primary meta-analysis of four publications, there was no evidence of an association between the use of topical beta-blockers and cardiovascular mortality (pooled HR estimate 1.10, 95% CI 0.84–1.36).

***Conclusion***: Topical beta-blockers do not appear to be associated with excess cardiovascular mortality. This evidence does not indicate that a change in current practice is warranted, although clinicians should continue to assess individual patients and their cardiovascular risk prior to commencing topical beta-blockers.

## Introduction

Topical beta-blockers remain one of the primary treatments for glaucoma.^1^ Previous studies have described associations between long-term use of topical beta-blockers and increased cardiovascular mortality.^2,3^ Topical medications can reach systemic concentrations, and up to 80% of timolol has been shown to be systemically absorbed.^4^ Furthermore, long-term treatment with timolol has been associated with dyslipidemia,^5–7^ also indicating systemic effects from topical treatment. Additional cardiovascular mechanisms include dysrhythmias and impaired cardiac output,^8^ although investigations into the association between topical beta-blocker use and cardiovascular mortality show conflicting results. The Blue Mountains Eye Study^2^ reported an increase in cardiovascular mortality in glaucoma patients using topical beta-blockers, whereas the Rotterdam Study^9^ found non-significant associations with long-term exposure. Given the significant global burden of cardiovascular disease, the widespread use of topical beta-blockers^10^ as a cost-effective treatment, and the availability of off-patent effective alternatives in the form of prostaglandin analogues,^11^ there is clear justification to explore the question further.

This study aimed to investigate the effects of topical beta-blockers on cardiovascular mortality. First, we examined the association in the European Prospective Investigation into Cancer (EPIC) Norfolk Cohort Study. Second, we conducted a systematic review of the literature and pooled our results with previously published data in a meta-analysis.

## Materials and methods

EPIC-Norfolk is a large, prospective, cohort study. All participants were invited to attend via their general practitioner (GP) between March 1993 and December 1997. The participation rate was 33%. As nearly all residents in the United Kingdom are registered with a GP through the National Health Service, general practice lists serve as population registers. Detailed methods have been published elsewhere.^12^ In brief, all persons aged 40–79 years in databases of the 35 participating general practices in the region were sent invitations (*N* = 77,360). Consent was received from 30,445 participants, of which 25,639 attended the first health examination.^12^ The Norfolk Local Research Ethics Committee approved the EPIC-Norfolk study, and all volunteers gave written informed consent.

We ascertained topical beta-blocker use through completion of a detailed health and lifestyle questionnaire and by asking participants to bring all previous prescriptions and medications they were taking at baseline to the health examination, where prescriptions and medicines were checked by trained nurses.^12^ All EPIC-Norfolk participants were followed up for mortality and flagged for death certification at the Office for National Statistics, with vital status ascertained on the whole cohort up until 30 June 2013.^12^ Coding of death certificates was performed by trained nosologists according to the International Classification of Diseases (ICD). Cardiovascular mortality was defined as any individual with ICD codes 410–448 (ICD 9th revision) or ICD codes I10–I79 (ICD 10th revision) listed as cause of death.

The design and characteristics of EPIC-Norfolk assessment methods for all covariables of interest have been described previously.^12^ The following covariables were measured through a self-reported questionnaire: History of angina, cholesterol, diabetes, education, glaucoma, myocardial infarction, stroke, hypertension, smoking and social class. Glaucoma ascertainment was also self-reported through direct questioning on the questionnaire. Systolic blood pressure (SBP) and diastolic blood pressure (DBP) were recorded during a health examination as the mean of two measurements taken from the right arm with the participant seated for 5 minutes, using an Accutorr Plus blood pressure monitor (Mindray, Huntingdon, UK).

Differences in characteristics of participants by exposure status were evaluated using Chi-squared and Welch’s unpaired T-tests. All participants with missing covariable data were included in the initial analyses, with a complete case analysis conducted on the final models. Associations between use of topical beta-blockers and cardiovascular mortality were assessed using Cox proportional hazard models. All analyses were adjusted for age and sex. Potential confounders were selected using univariable comparisons of covariables, and variables which were significantly associated with both exposure and outcome were included. We also included covariables based on *a priori* assumptions of known confounders in the final Cox model. Raised cholesterol was considered to be a potential mediator on the causal pathway^5–7^ and therefore not included in the final model. A subgroup analysis was performed to look at the association between topical beta-blocker exposure and cardiovascular mortality in only participants with self-reported glaucoma. Multiple sensitivity analyses were carried out to explore the robustness of our findings. The effect of different covariables, glaucoma status, cholesterol as a confounder rather than a mediator, whether having manifest cardiovascular disease might alter exposure patterns and introduce reverse causation were explored. No adjustments were made for change in exposure status over the course of the study period. This allowed direct comparison with methods from the Blue Mountains Eye Study^2^ and Rotterdam Study^9^. However, as glaucoma is an age-related disease,^13^ and given the older age range within the EPIC-Norfolk cohort,^12^ it is probable that further incident cases of glaucoma and thus topical beta-blocker exposure occurred. Therefore, we used census-derived population data, together with epidemiological models to estimate the potential impact of incident glaucoma during the follow-up period. We also examined the association between topical beta-blocker use and cardiovascular mortality at the second health check (1998–2000) to explore the effect of changing treatment during the follow-up period.

### Systematic literature review

A literature-based meta-analysis was performed to look for published evidence on the association between topical beta-blockers and cardiovascular mortality. The search strategy was performed within PubMed (National Center for Biotechnology Information, USA).

The following terms were used in the literature search strategy in July 2014;

(1) topical[All Fields], (2) “adrenergic beta-antagonists” [Pharmacological Action] OR “adrenergic beta-antagonists”[MeSH Terms] OR (“adrenergic” [All Fields] AND “beta-antagonists”[All Fields]) OR “adrenergic beta-antagonists” [All Fields] OR (“beta” [All Fields] AND “blockers” [All Fields]) OR “beta blockers”[All Fields], (3) “glaucoma”[MeSH Terms] OR “glaucoma”[All Fields], (4) “mortality”[Subheading] OR “mortality”[All Fields] OR “mortality”[MeSH Terms], (5) (“cardiovascular system”[MeSH Terms] OR (“cardiovascular”[All Fields] AND “system”[All Fields]) OR “cardiovascular system”[All Fields] OR “cardiovascular”[All Fields]) AND (“mortality”[Subheading] OR “mortality”[All Fields] OR “mortality”[MeSH Terms]), (6), (1) AND (2), (7) (3) OR (6), (8) (4) OR (5). The search (7) AND (8) returned 278 publications.

Publications were reviewed if they reported associations between topical beta-blocker use and cardiovascular mortality. No exclusion criteria were applied to language or journal publication date. Publications were excluded if they were non-human studies, *in-vitro* experiments, ecological studies, case series, case reports, opinion pieces, letters, did not look at topical beta-blockers as an exposure and did not look at cardiovascular mortality as an outcome. Publications were additionally excluded if they were reviews of published associations with no new contributing data.

**Figure 1.**
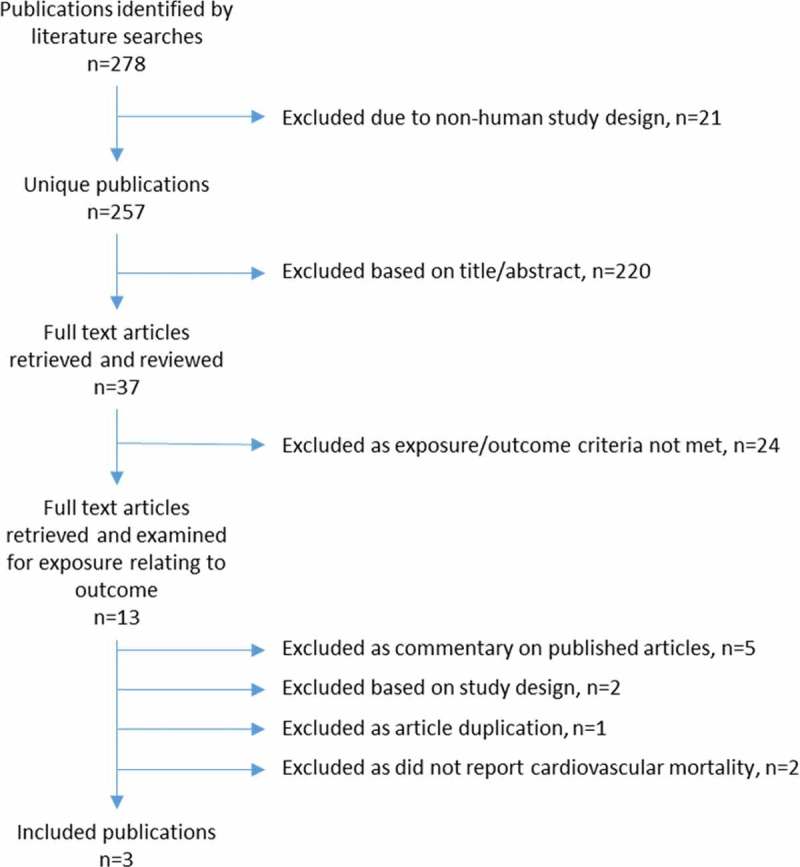
Flowchart of the literature review on the association between topical beta-blockers and cardiovascular mortality illustrating the exclusion process of retrieved publications.

The exclusion process is detailed in Figure 1. The resulting three articles were all cohort studies and are summarized in Table 1.

**Table 1.** Summary of three published studies reporting associations between topical beta-blocker use and cardiovascular mortality.

Author	Study design	Participants	Exposure	Exposure assessment	Outcome	Outcome assessment	Covariables	Results
Lee et al.^2^	Cohort study	*N* = 3654, 49–97 years, Blue Mountains, Australia	Glaucoma status, baseline medications	Detailed baseline eye examinations using Humphrey 30-2 visual field test for glaucoma. Face-to-face interviews for medication history.	All-cause mortality and cause of death	Matching participant demographic data to the Australian National Death Index using a probabilistic linkage package. Non-exact matches examined manually and corroborated with information from family members.	Age, sex, hypertension, diabetes, heart attack, angina, stroke, cancer, smoking, alcohol consumption, oral beta-blocker use, myopia, cataract	No association between all-cause mortality and glaucoma. Increased risk of cardiovascular mortality in those with glaucoma in <75 years cohort and previously diagnosed glaucoma (RR 2.78 95% CI 1.20–6.47). Further stratified analysis showed increased risk in those with previously diagnosed glaucoma (RR 1.85, 95% CI 1.12–3.04), more so in those using topical timolol as part of a trend analysis (RR 2.14, 95% CI 1.18–3.89). No association of increased risk of CVD shown in the 88 baseline users of timolol vs non-users (RR 1.47, 95% CI 0.90–2.41).
Muskens et al.^9^	Cohort study	*N* = 3842, 55+ years, Rotterdam, Netherlands	Topical beta-blockers	All pharmacies in region using fully automated single network on use, date and type of beta-blocker. Matched to participants.	All-cause mortality and cause of death	From municipal registry, vital status checked on bi-weekly basis along with GP reported deaths. All deaths checked by specially trained personnel. CVD deaths according to relevant ICD-10 codes. Mortality data in 100% of cases. Certainty of cause in 84% of cases.	Age, sex, smoking, hypertension, diabetes, angina	No association between topical beta-blocker use and all-cause mortality (HR 0.94, 95% CI 0.71–1.25) or cardiovascular mortality (HR 1.02, 95% CI 0.56–1.86).
Wu et al.^3^	Cohort study	*N* = 4092, 40–84 years, Barbados	Glaucoma status, baseline medications	Detailed baseline eye examination including Humphrey automated perimetry and detailed visual field assessment.	All-cause mortality and cause of death	Verified from death certificates held at Ministry of Health.	Age, sex, diabetes, hypertension, CVD, stroke	No association between OAG and increased mortality. In those with OAG at baseline, those treated with timolol had higher cardiovascular mortality (RR 1.91, 95% CI 1.04–3.50). However when comparing CVD mortality in timolol users to all non-users, no increased risk of cardiovascular mortality was observed (RR 1.58, 95% CI 0.97–2.58).

CI, confidence interval; CVD, cardiovascular disease; GP, general practitioner; HR, hazard ratio; ICD-10, international classification of diseases 10th revision; OAG, open-angle glaucoma; RR, relative risk.

A random effects model was used to pool the results and accommodate between-study heterogeneity. Between-study heterogeneity was analyzed using the I-squared test statistic. All analyses were carried out in Stata 12.1 (StataCorp, College Station, TX, USA).

## Results

A total of 25,639 participants with a mean age of 59.2 years (standard deviation, SD, 9.32 years) were included in this analysis. Participants were followed up for a median of 17.0 years and a total of 407,524 person-years. During the follow-up period, 6525 individuals died (25.4%) of which 2158 deaths (33.1%) were attributed to cardiovascular disease. A total of 19,114 individuals were alive at the time of follow-up. Topical beta-blockers were used by 179 individuals (0.7%) of whom 82 (45.8%) had died during follow-up. Of these 36 (20.1%) participants who used topical beta-blockers died of cardiovascular causes.

Table 2 details the baseline characteristics of the cohort. Those using topical beta-blockers were older at baseline, with a mean difference of 7.4 years (95% confidence interval, CI, 6.2–8.5 years). Just over half (57.8%) of participants using topical beta-blockers were male. Significant differences in the proportions of those with diabetes, angina, myocardial infarction, stroke, self-reported hypertension, raised cholesterol, and raised SBP at baseline were observed, with higher proportions in those using topical beta-blockers. A significantly higher proportion of ever smokers were also seen in topical beta-blocker users. Non-significant differences in social class, education level, and raised DBP were observed between exposure groups. The proportion of participants using topical beta-blockers with self-reported glaucoma was predictably high, with 89.9% of users reporting glaucoma. Of the 514 participants with glaucoma, 353 (68.7%) were not using topical beta blockers compared with 161 (31.3%) who were.

**Table 2.** Comparison of baseline characteristics between exposure groups in participants in the European Prospective Investigation into Cancer Norfolk Cohort Study (EPIC-Norfolk, 1993–2013).

Baseline characteristic	No topical beta-blocker use (*n* = 25,460)	Topical beta-blocker use (*n* = 179)	*p*-value for difference between exposure groups^a^
Age, mean (SD), years	59.2 (9.3)	66.6 (7.6)	<0.001
Sex, % female	54.8	42.2	<0.001
Diabetes, %	2.3	5.6	0.003
Myocardial infarction, %	3.1	10.1	<0.001
Angina, %	5.1	11.7	<0.001
Self-reported hypertension, %	14.2	25.7	<0.001
Self-reported raised cholesterol level, %	8.2	14.0	0.004
Stroke, %	1.4	3.4	0.028
Glaucoma, %	1.4	89.9	<0.001
Systolic blood pressure, mean (SD) mmHg	135.0 (18.4)	141.0 (19.6)	<0.001
Diastolic blood pressure, mean (SD) mmHg	82.5 (11.3)	83.7 (11.8)	0.178
Smoking status, % current/ever	54.0	61.8	0.038
Social class, % non-manual	60.0	66.5	0.080
Education level, % higher	52.9	49.7	0.397

^a^Dichotomous covariables; *p*-value for independence calculated using Chi-squared test. Continuous variables; *p*-value for difference calculated from unpaired Welch’s T-test. SD, standard deviation.

Table 3 details the results of the Cox proportional hazards analysis for the complete cohort. Topical beta-blocker use was not associated with cardiovascular mortality (hazard ratio, HR, 1.12, 95% CI 0.80–1.56) when adjusted for age and sex. Table 3 also presents the maximally adjusted model; no significant association was found between use of topical beta-blockers and cardiovascular mortality (HR 0.93, 95% CI 0.67–1.30).

**Table 3.** Associations between topical beta-blocker use and cardiovascular mortality in the European Prospective Investigation into Cancer Norfolk Cohort Study (EPIC-Norfolk, 1993–2013).

	Hazard Ratio (95% confidence interval)	*p*-value
Model 1: Cardiovascular mortality^a^		
Topical beta-blocker use^b^	1.116 (0.802–1.553)	0.513
Age, per year	1.190 (1.182–1.198)	<0.001
Sex, female	0.491 (0.450–0.535)	<0.001
Model 2: Cardiovascular mortality^a^		
Topical beta-blocker use^+^	0.931 (0.668–1.299)	0.677
Age, per year	1.178 (1.170–1.187)	<0.001
Sex, female	0.590 (0.537–0.648)	<0.001
Diabetes	2.118 (1.783–2.516)	<0.001
Myocardial infarction	2.287 (1.968–2.659)	<0.001
Angina	1.294 (1.128–1.486)	<0.001
Stroke	2.272 (1.869–2.761)	<0.001
Systolic hypertension	1.579 (1.432–1.740)	<0.001
Social class, non-manual	0.854 (0.782–0.933)	<0.001
Smoking status	1.339 (1.216–1.475)	<0.001

^a^6525 deaths (25.4%); 2158 deaths due to CVD.

^b^179 participants on topical beta-blockers, of whom 82 (45.8%) died; 36 from CVD. Model 1 adjusted for age and sex (*N* = 25,639), model 2 adjusted for age, sex, diabetes, myocardial infarction, angina, stroke, hypertension, social class, and smoking status, using cox proportional hazards model (*N* = 24,724 due to missing covariables). CVD, cardiovascular disease.

At baseline, 514 participants (2.0%) had glaucoma of which 229 (44.6%) died during follow-up. Of these 92 participants (17.9%) died of cardiovascular causes. Overall, 285 participants with glaucoma were alive at the end of the study. Table 4 shows the results of the Cox proportional hazards analysis for glaucoma patients adjusted for age and sex. Comparing participants reporting glaucoma and using topical beta-blockers to those reporting glaucoma not using topical beta-blockers, there was no association with cardiovascular mortality when adjusted for age and sex (HR 0.76, 95% CI 0.49–1.18) or when fully adjusted (HR 0.89, 95% CI 0.56–1.40). Results from the sensitivity analyses did not indicate substantial effects to the final model from additional covariables, inclusion of cholesterol or higher levels of beta-blocker use with follow-up time. Sensitivity analysis to estimate the impact of incident glaucoma did not indicate a substantial effect from incident glaucoma cases. Altogether, 52,986 individuals aged over 40 years currently live in the catchment area of the initial EPIC-Norfolk recruitment. Using the UK National Eye Health Epidemiological Model,^14^ numbers with incident glaucoma were calculated from this cohort and applied to the aging EPIC-Norfolk cohort. Non-conservative models predicted a 1.56% incident rate of glaucoma. Applied to the baseline population excluding those with self-reported glaucoma, this predicted an additional 392 cases of incident glaucoma over the study period. Trends in glaucoma medication prescribing have changed with time, where prostaglandins are the preferred medication^15^ and beta-blocker prescription has dropped by a factor of four. Applying an exaggerated 25% prevalence of topical beta-blocker exposure to these incident cases^15^ would lead to an additional 98 topical beta-blocker exposures in the EPIC-Norfolk cohort. Applying an exaggerated death rate of 50% to those newly diagnosed with glaucoma on topical beta-blockers, would add an additional 49 deaths. Assuming 30% died from cardiovascular causes compared to the 20.1% observed in the present study, along with adjustments in exposure status in baseline users through application of historic trends in prescribing,^15^ did not yield significant associations of cardiovascular mortality (HR 1.30, 95% CI 0.98–1.70, *p* = 0.06). This would suggest the findings of this study are robust to changes in exposure status over time.

**Table 4.** Associations between topical beta-blocker use and cardiovascular mortality in 514 participants with self-reported glaucoma in the European Prospective Investigation into Cancer Norfolk Cohort Study (EPIC-Norfolk, 1993–2013).

	Hazard Ratio (95% confidence interval)	*p*-value
Model 1: Cardiovascularmortality in those withglaucoma		
Topical beta-blocker use	0.757 (0.485–1.180)	0.219
Age, per year	1.203 (1.150–1.257)	<0.001
Sex, female	0.598 (0.394–0.907)	0.016
Model 2: Cardiovascularmortality in those withglaucoma		
Topical beta-blocker use	0.885 (0.557–1.401)	0.605
Age, per year	1.195 (1.140–1.252)	<0.001
Sex, female	0.765 (0.484–1.210)	0.252
Diabetes	2.550 (1.376–4.729)	0.003
Myocardial infarction	1.119 (0.519–2.413)	0.775
Angina	1.735 (0.905–3.327)	0.097
Stroke	5.119 (2.316–11.316)	<0.001
Hypertension	1.015 (0.608–1.694)	0.956
Social class, non-manual	0.743 (0.465–1.189)	0.216
Smoking status	1.246 (0.756–2.054)	0.389

Model 1 adjusted for age and sex, model 2 adjusted for age, sex, diabetes, myocardial infarction, angina, stroke, hypertension, social class, and smoking status, using cox proportional hazards model.

**Figure 2.**
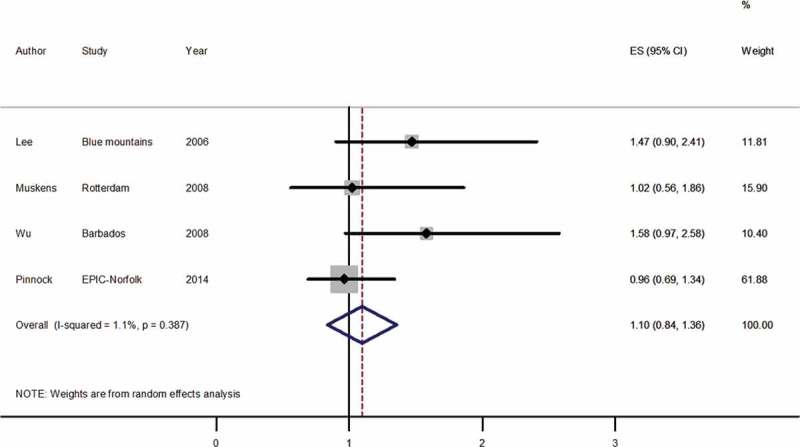
Forest plot of random effects meta-analysis examining effect of topical beta-blocker use on cardiovascular mortality. ES, effect size; CI, confidence interval; Blue Mountains, Blue Mountains Eye Study; Rotterdam, Rotterdam Study; Barbados, Barbados Eye Studies; EPIC-Norfolk, European Prospective Investigation into Cancer Norfolk Cohort Study.

A total of three studies,^2,3,9^ that looked at topical beta-blockers and cardiovascular mortality were identified from the systematic literature search. Figure 2 presents results from the meta-analysis for the effect of topical beta-blockers on cardiovascular mortality, regardless of glaucoma status. Between-study heterogeneity using the I^2^ statistic was 1.1% (*p* = 0.387). Topical beta-blocker use was not associated with cardiovascular mortality (pooled HR estimate 1.10, 95% CI 0.84–1.36).

## Discussion

In this prospective cohort study of 25,639 participants, topical beta-blocker use was not associated with increased cardiovascular mortality. Furthermore, in the 514 participants within the cohort who had self-reported glaucoma, we did not detect an association between the use of topical beta-blockers and a higher risk of cardiovascular mortality. There was also no evidence of an association between the use of topical beta-blockers and cardiovascular mortality. These results do not support higher cardiovascular mortality from topical beta-blocker use. However, the current study is based on self-reported glaucoma, which is a key limitation.

Results from this EPIC-Norfolk study are in agreement with the Rotterdam Study^9^ but differ from the Blue Mountains Eye Study^6^ and Barbados Eye Studies.^3^ In the Blue Mountains Eye Study, the association between topical beta-blockers and cardiovascular mortality was only seen in participants using timolol with glaucoma, compared to those not using topical timolol without glaucoma. The effect on cardiovascular mortality was not shown in baseline users of timolol before stratification by glaucoma status (relative risk, RR, 1.47, 95% CI 0.90–2.41). In baseline timolol users in the Barbados Eye Studies, there was also no association shown between timolol use and cardiovascular mortality (RR 1.58, 95% CI 0.97–2.58). The association was only seen when compared within users diagnosed with open-angle glaucoma. The primary meta-analysis used these summary statistics in order to compare like with like and did not show an association between topical beta-blocker use and cardiovascular mortality. Subgroup analysis of participants with self-reported glaucoma in the present study found no association between exposure to topical beta-blockers and cardiovascular mortality (HR 0.89, 95% CI 0.56–1.40).

There is well-established literature on the protective effects of oral beta-blockers. Multiple randomized controlled trials and systematic reviews have found that use of systemic beta-blockers decreases mortality, improves survival, and is cardio-protective.^16–20^ However, the majority of these studies have shown the protective effect only in participants who are post myocardial infarction or with established cardiovascular disease. These observed benefits may not translate to people with no pre-existing cardiovascular disease. The main cardiovascular effects of beta-blockers include reduction of heart rate, myocardial contractile force, cardiac output and peripheral vasoconstriction. Unwanted side effects include bradycardia and exacerbation of heart failure in susceptible patients, and these complications form a potential mechanism for increased risk of cardiovascular mortality from topical beta-blocker use, although there is little observed evidence of this beyond case reports.^8^ Randomized controlled trials have shown that topical beta-blockers may have an adverse effect on lipid profiles. A review of timolol, the most common topical beta-blocker linked to adverse lipid profiles, found that when given systemically, timolol reduced mortality after myocardial infarction^21^ indicating that any deleterious effects on serum lipids did not attenuate its protective effect on the heart.

The strengths of this study lie in its study design; EPIC-Norfolk is a long-term prospective cohort study. Other strengths include the long period of follow-up, the number of participants, and the high number of incident cardiovascular mortality events, enabling high statistical power. The large number of covariables measured at baseline allowed for adjustment of multiple confounders in the analysis and the assessment of the robustness of the findings across different analytical variations. A validation study^22^ has confirmed accuracy of cardiovascular mortality in EPIC-Norfolk, with 38 of 39 cases confirmed as correct. Last, the linking of outcome data through the Office for National Statistics death register allowed for complete ascertainment of outcome status in this study, regardless of whether the participant was lost to follow-up. This eliminated attrition bias.

There were several key limitations of the study. There was limited power within the subgroup analysis of glaucoma users whose baseline cohort was small (*n* = 514). This was further reduced after excluding deaths not from cardiovascular causes (*n* = 377) and may have allowed type II error to occur. There may be a degree of healthy-volunteer bias occurring. This is suggested by the considerably lower proportion of current and ex-smokers, and a higher proportion of never smokers compared to the rest of England. This may also have contributed to the relatively low proportions of cardiovascular deaths in this older population. The study only assigned participants to the exposed category if topical beta-blockers were used before or at baseline and no adjustments were made for changes in exposure status with time. Additionally, it was not possible to ascertain compliance of individuals exposed to topical beta-blockers, who may have filled prescriptions they were not taking. This may have resulted in an underestimate of effect. Our methods are in line with the previous studies examined in the meta-analysis, and allowed for direct comparisons. We did, however, examine the impact of incident glaucoma and did not detect a significant effect. Publication bias was possible in the meta-analyses, but the use of a funnel plot was of little value due to the number of studies identified. It is plausible that there are smaller studies that have unpublished non-significant results that would strengthen the absence of an association between topical beta-blocker use and cardiovascular mortality and favor the null hypothesis.

We did not examine the relationship between glaucoma diagnosis and mortality as it was beyond the scope of this study. It is possible that topical beta-blocker use may mask heterogeneous pathophysiological processes that underpin both glaucoma and ocular hypertension. Both conditions are treated with topical beta-blockers and display different associations with hypertension and diabetes.^23–26^ This is particularly relevant to this study as we used self-reported glaucoma, where participants could report a glaucoma diagnosis even if they had ocular hypertension. Furthermore, up to 50% of glaucoma in the community can be undiagnosed.^27,28^ These considerations would lead to a measurement error in exposure ascertainment, and although likely to contribute to a null finding, the exact impact is unknown.

In conclusion, no association was observed between topical beta-blocker use and cardiovascular mortality in all participants or in participants with self-reported glaucoma. A meta-analysis with other large population-based studies showed no association in the pooled estimates between topical beta-blockers and cardiovascular mortality, both for all participants and for only participants with glaucoma. However, further studies are required that allow for adjustment for changes in exposure to topical beta-blockers. We have found no further evidence to suggest that patients using topical beta-blockers experience excess cardiovascular mortality, and no indications that current clinical practice requires revision. Clinicians should continue to exercise caution when assessing individual glaucoma patients and their cardiovascular risk prior to commencing topical beta-blockers.
